# Classification of pig calls produced from birth to slaughter according to their emotional valence and context of production

**DOI:** 10.1038/s41598-022-07174-8

**Published:** 2022-03-07

**Authors:** Elodie F. Briefer, Ciara C.-R. Sypherd, Pavel Linhart, Lisette M. C. Leliveld, Monica Padilla de la Torre, Eva R. Read, Carole Guérin, Véronique Deiss, Chloé Monestier, Jeppe H. Rasmussen, Marek Špinka, Sandra Düpjan, Alain Boissy, Andrew M. Janczak, Edna Hillmann, Céline Tallet

**Affiliations:** 1 Institute of Agricultural Sciences, ETH Zurich, Universitätsstrasse 2, 8092 Zürich, Switzerland; 2 Behavioural Ecology Group, Section for Ecology and Evolution, Department of Biology, University of Copenhagen, 2100 Copenhagen, Denmark; 3 Department of Ethology, Institute of Animal Science, 104 01 Prague, Czechia; 4 Department of Zoology, Faculty of Science, University of South Bohemia, 370 05 Č. Budějovice, Czechia; 5 Institute of Behavioural Physiology, Research Institute for Farm Animal Biology (FBN), 18196 Dummerstorf, Germany; 6 Department of Agricultural and Environmental Sciences, Università Degli Studi Di Milano, Milano, Italy; 7 Faculty of Veterinary Medicine, Norwegian University of Life Sciences, Universitetstunet 3, 1433 Ås, Norway; 8 PEGASE, INRAE, Institut Agro, 35590 Saint Gilles, France; 9 University of Clermont Auvergne, INRAE, VetAgro Sup, UMR Herbivores, 63122 Saint-Genès Champanelle, France; 10 Bureau ETRE, 63210 Bravant, Olby France; 11 Center for Coastal Research, University of Agder, 4604 Kristiansand, Norway; 12 Center for Artificial Intelligence Research, University of Agder, 4604 Kristiansand, Norway; 13 Faculty of Agrobiology, Food and Natural Resources, Czech University of Life Sciences, 165 21 Prague, Czechia; 14 Animal Husbandry and Ethology, Albrecht Daniel Thaer-Institut, Faculty of Life Sciences, Humboldt-Universität Zu Berlin, Philippstrasse 13, 10115 Berlin, Germany; 15 School of Engineering and Applied Sciences, Harvard University, Cambridge, MA USA

**Keywords:** Animal behaviour, Zoology

## Abstract

Vocal expression of emotions has been observed across species and could provide a non-invasive and reliable means to assess animal emotions. We investigated if pig vocal indicators of emotions revealed in previous studies are valid across call types and contexts, and could potentially be used to develop an automated emotion monitoring tool. We performed an analysis of an extensive and unique dataset of low (LF) and high frequency (HF) calls emitted by pigs across numerous commercial contexts from birth to slaughter (7414 calls from 411 pigs). Our results revealed that the valence attributed to the contexts of production (positive versus negative) affected all investigated parameters in both LF and HF. Similarly, the context category affected all parameters. We then tested two different automated methods for call classification; a neural network revealed much higher classification accuracy compared to a permuted discriminant function analysis (pDFA), both for the valence (neural network: 91.5%; pDFA analysis weighted average across LF and HF (cross-classified): 61.7% with a chance level at 50.5%) and context (neural network: 81.5%; pDFA analysis weighted average across LF and HF (cross-classified): 19.4% with a chance level at 14.3%). These results suggest that an automated recognition system can be developed to monitor pig welfare on-farm.

## Introduction

Animal emotions, defined as short-term intense affective reactions to specific events, have been of increasing interest over the last few decades, especially because of the growing concern for animal welfare^1^. Research in animals confirms that emotions are not automatic and reflexive processes, but can rather be explained by elementary cognitive processes^2^. This line of thinking suggests that an emotion is triggered by the evaluation that an individual makes of its environmental situation^3^. The dimensional approach, that categorizes emotions according to their two main dimensions—their valence (pleasant/positive versus unpleasant/negative) and their arousal (bodily activation) -, offers a good framework to study emotional experiences in animals^4^.

Emotions can be expressed through visual, olfactory, and vocal signals to allow the regulation of social interactions^5,6^. During vocal production, emotions can influence the physiological structures that are the basis of sound production at several levels (lungs, larynx and vocal tract), thus modifying sound structure itself (e.g. sound duration, amplitude, fundamental frequency, energy distribution)^7,8^.

Due to the impact of emotions on vocalization, the analysis of vocal expression of emotions is increasingly being considered as an important non-invasive tool to assess the affective aspects of animal welfare^9,10^. In the last decade, it has been shown that vocalizations of various animal species produced in specific emotional contexts and/or physiological states display specific acoustic characteristics^10–12^. Furthermore, systems for automatic acoustic recognition of physiological and stress states have already been developed for cattle^13,14^ and pigs^15^. These systems detect specific sounds (e.g. high-frequency calls), which may serve as first indicators of impaired welfare^16^. Nevertheless, the real challenge remains to create a tool that can accurately identify the emotional states of the animals based on real-time call detection and classification in various environments.

Up to now, studies on vocal indicators of emotions have often been restricted to specific call types produced by animals of a given age, living in a specific environment and experiencing a limited number of well-defined situations^11^. Such factors create a high degree of between-study variance, which must be accounted for in a system aiming at the identification of global states in diverse contexts. Additional changes in the parameters derived from acoustic recordings are induced by the ‘acoustic environment’, due to different levels of noise (e.g. ventilation indoors, other animals) and reverberation depending on the properties of surrounding surfaces. Therefore, a cross-context validation is needed to separate emotion-related variance from context-related variance, in order to identify reliable indicators of emotions.

In the domestic pig, a species in which vocal communication is highly developed, acoustic features of vocalizations vary according to the context of production^17^. Part of this acoustic variance may reflect the emotional dimensions of valence and arousal. However, the relationship between valence and vocal expression is complex because pigs use a repertoire of several call types across contexts, and the acoustic parameters may change differently according to valence or arousal in different call types^18,19^. Specifically, previous research has shown that domestic pig vocalizations can be distinguished into high-frequency (HF) and low-frequency calls (LF), with 2–3 less distinct subcategories within each of the two major types^17^. HF calls (screams, squeals) are common in negative contexts, while LF calls (grunts) prevail in neutral and positive situations^17^. Thus, HF calls could be used as an indicator of negative affective valence^15^. Yet, there is also a large within call-type variation (e.g. duration, formants, energy distribution^18–21^) that could be used as additional way to assess emotional valence and arousal, and to identify the contexts in which the calls were emitted.

The aim of this study was to identify the features of pig vocalizations that are most indicative of emotional state and context, in order to thereby provide a basis for the development of a tool able to automatically assess valence and detect particular situations from real-time acoustic input. Towards this aim, we performed an analysis of an extensive and unique dataset of vocalizations emitted across many different situations from the birth to slaughter of commercial pigs (7414 calls produced by 411 pigs). We first tested how specific vocal parameters change as a function of the valence attributed to the contexts, and as a function of the contexts themselves. We then tested two different automated methods of classifying the calls; a permuted discriminant function analysis based on a limited number of extracted vocal parameters, and an image classification neural network based on spectrograms of the calls. The efficacy of these two methods for classifying calls to the correct valence and context of production is discussed with regards to the potential for building an automated on-farm real-time classification tool.

## Results

In total, we analyzed 7414 HF and LF calls produced by 411 pigs in 19 different context categories (Supplementary Table S1).

### Changes to specific vocal parameters

Four vocal parameters (call duration [Dur], amplitude modulation rate [AmpModRate], spectral center of gravity [Q50%] and mean Wiener Entropy [WienEntropy]) were selected on the basis of a Principal Component Analysis for inclusion in Linear Mixed-Effects Models (LMM) to investigate the effects of the emotional valence (positive or negative) and the context (19 context categories) on the vocalizations (Supplementary Table S1).

#### Effects of the valence

**Figure 1:**
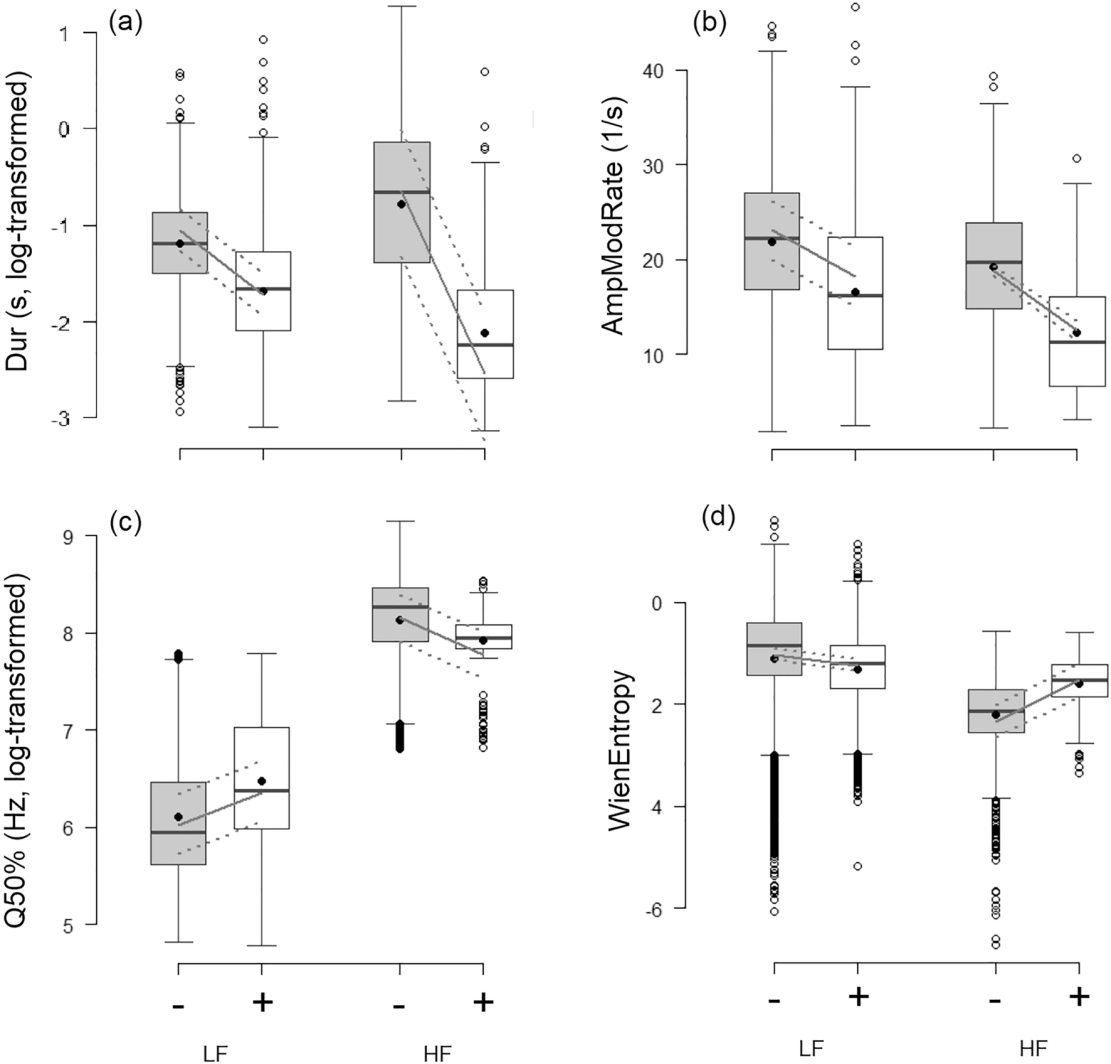
Effect of the valence on the vocal parameters. (**a**) Call duration (Dur), (**b**) Amplitude modulation rate (AmpModRate), (**c**) Spectral center of gravity (Q50%) and (**d**) Wiener entropy (WienEntropy), as a function of the valence (“ − ” = negative (grey); “ + ” = positive (white)) and call type (“LF” = low-frequency calls; “HF” = high-frequency calls). Boxplots: the horizontal line shows the median, the box extends from the lower to the upper quartile and the whiskers to 1.5 times the interquartile range above the upper quartile or below the lower quartile, and open circles indicate outliers and black circles the mean; the grey lines show the model estimates (continuous line) and 95% confidence intervals (dashed lines). All comparisons between negative and positive valence, for each call type, were significant (LMM: *p* ≤ 0.001).

All LMMs revealed an effect of the valence for both low-frequency calls (LF) and high-frequency calls (HF) (Fig. 1; *p* ≤ 0.001 for all models). Both types of calls were shorter (Dur; *R*^*2*^_GLMM(m)_: LF = 0.27, HF = 0.30; Fig. 1a) and had fewer amplitude modulations (AmpModRate; *R*^*2*^_GLMM(m)_: LF = 0.09, HF = 0.08; Fig. 1b) in positive contexts than in negative ones. By contrast, the effect of valence on Q50% and WienEntropy depended on the call type. Q50% (Fig. 1c) measured in LF calls was higher in positive contexts compared to negative contexts, while the opposite was found for HF calls (*R*^*2*^_GLMM(m)_: LF = 0.05, HF = 0.04). WienEntropy (Fig. 1d) measured in LF calls was lower in positive contexts, indicating more tonal calls, compared to negative contexts, while the opposite was found for HF calls (*R*^*2*^_GLMM(m)_: LF = 0.01, HF = 0.10).

#### Effects of the context category

The context category affected Dur (*R*^*2*^_GLMM(m)_: LF = 0.38, HF = 0.52), AmpModRate (*R*^*2*^_GLMM(m)_: LF = 0.24, HF = 0.13), Q50% (*R*^*2*^_GLMM(m)_: LF = 0.34, HF = 0.08), and WienEntropy (*R*^*2*^_GLMM(m)_: LF = 0.16, HF = 0.17) for both call types (*p* < 0.001 for all models; see Supplementary Figure S1-S4 for the values related to the 19 context categories).

### Automated classification

In order to evaluate if pig calls could be automatically classified to the correct valence and/or context of production, we performed a permuted discriminant function analysis (pDFA) and a machine learning algorithm, based on an image classifying neural network.

#### Permuted discriminant function analysis

We first proceeded to a pDFA based on the four parameters we selected for inclusion in our LMMs (Dur, AmpModRate, Q50%, and WienEntropy). When considering non-cross-classified calls, both LF and HF calls could be classified to the correct valence (weighted average across LF and HF: correct classification = 85.2%; chance level = 55.87%) or context category of production (correct classification = 24.4%; chance level = 15.48%) by the pDFA above chance levels (*p* = 0.001 for all; Table 1). Percentages of cross-classified calls (i.e. not used for deriving the discriminant functions) were, however, much lower. With a cross-classification, both LF and HF calls could still be classified to the correct context category of production by the pDFA slightly above chance levels (weighted average across LF and HF: correct classification = 19.5%; chance level = 14.3%; *p* ≤ 0.017; Table 1). Yet, only LF (p = 0.004), but not HF calls (p = 0.169), could be classified to the correct valence above chance level (weighted average across LF and HF: correct classification = 61.7%; chance level = 50.5%; Table 1).

**Table 1:** Correct classification of calls according to the valence and context of production by the pDFA.

	Valence		Context	
	LF	HF	LF	HF
No. valence/contexts category	2	2	19	16
No. individuals	392	261	392	261
Total No. calls	5391	1832	5391	1832
No. calls selected	236	80	597	355
Correctly classified (%)	81.32	96.59	20.24	36.6
Chance level (%)	54.62	59.55	12.31	24.81
*P* value for classified	**0.001**	**0.001**	**0.001**	**0.001**
Correctly cross-classified (%)	61.25	63.18	16.20	29.40
Chance level for cross-classified (%)	50.55	50.37	11.28	23.11
Relative cross-classification level	1.21	1.25	1.44	1.27
*P* value for cross-classified	**0.004**	0.169	**0.003**	**0.017**

Results of the permuted discriminant function analysis (pDFA) for low-frequency calls (LF) and high-frequency calls (HF); number of valence or contexts included, number of individuals, total number of calls, number of calls selected, percentage of calls classified and cross-classified to the correct valence or context, and corresponding chance level (expected percentage of correctly classified calls based on the permutation test, averaged across the permutations), relative classification (percentage of calls cross-classified/chance level), and *p* value. The analysis was performed on the entire dataset, after excluding missing data (Sample size: calls in which AMRate could not be measured = 191; calls in the entire dataset = 7414; calls included for this analysis = 7223). Significant *p* values appear in bold.

#### Neural network

We tested a second automated classification approach, using a convolutional neural network and spectrograms created from the complete vocalizations. This method showed an accuracy of 91.5 ± 0.3% for classifying vocalizations according to valence, and of 81.5 ± 0.3% for classifying vocalizations according to context (Table 2).

**Table 2:** Performance statistics for neural networks trained on valence and context of production.

	Valence	Context
Accuracy	0.915 ± 0.003	0.815 ± 0.003
Precision	0.919 ± 0.005	0.815 ± 0.003
Recall	0.912 ± 0.003	0.813 ± 0.003
F1 score	0.916 ± 0.003	0.812 ± 0.003

For the binary valence classifier (2 classes: positive and negative), the following statistics were computed using the binary precision, recall, and F1 score formulas while treating positive valence labels as positive. For the imbalanced multi-class context classifier (19 classes, Supplementary Table S1), the following statistics were calculated as weighted averages across the classes. From the 10 trials, the mean accuracy, precision, recall, and F1 scores of the classifiers are listed. The uncertainty value is calculated across 10 trials.

**Figure 2:**
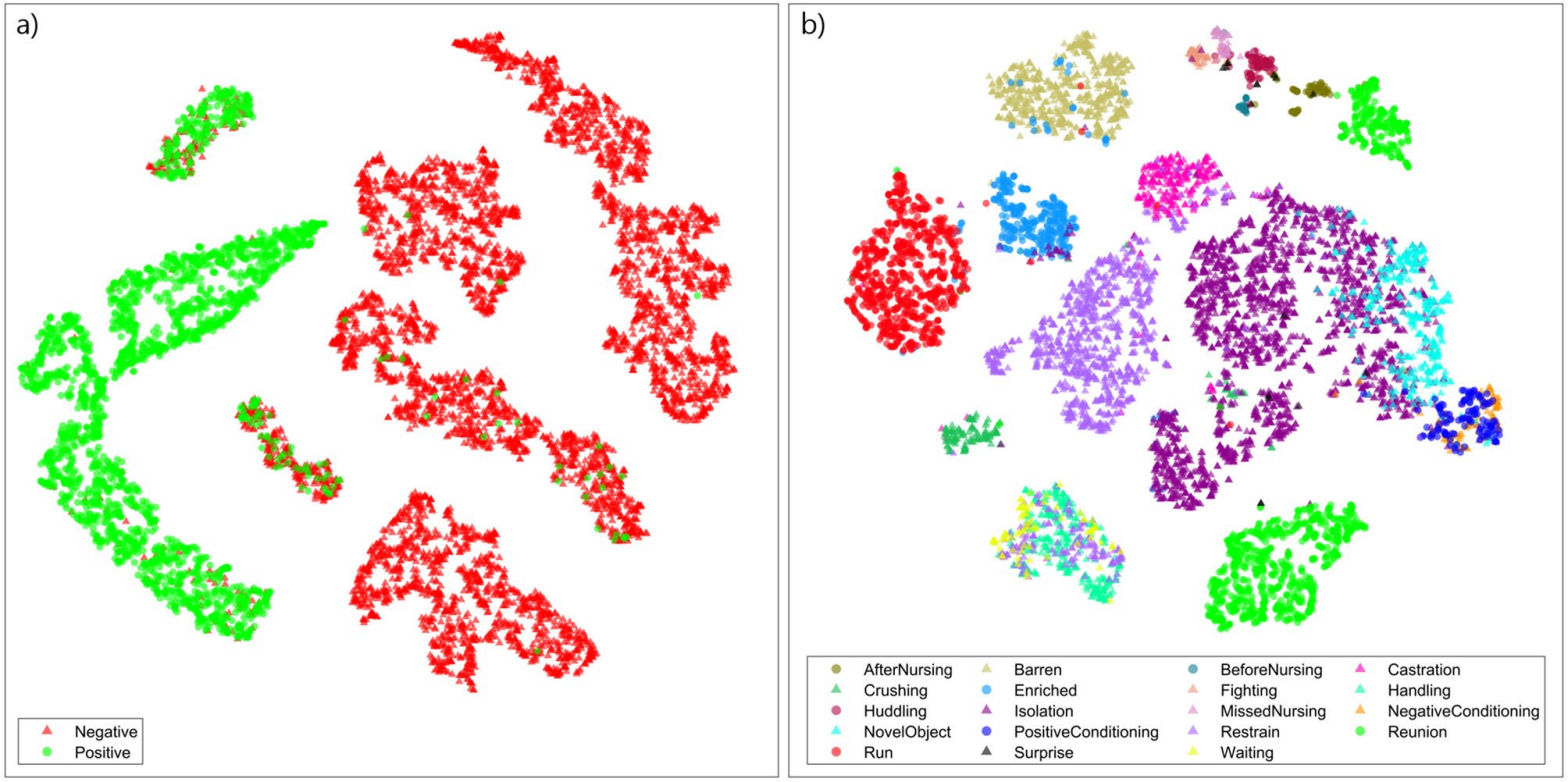
Classification of calls to the valence and context of production based on t-SNE. t-SNE embedding of (**a**) valence (embedding perplexity = 50) and (**b**) context (embedding perplexity = 20) classifying neural network’s last fully connected layer activations for each spectrogram (t-SNE plots visualize the probability that two points are neighbors in an original multivariate space). Triangles indicate negative valence vocalizations, while circles indicate positive ones (see Supplementary Text for more information on the settings used for this figure).

To further investigate how the neural network parsed the vocalizations, the last fully connected layer of the neural networks (one for valence, another for context) was analyzed by a dimensionality reduction machine learning algorithm called t-distributed Stochastic Neighbors Embedding (t-SNE)^22^. By applying t-SNE, visualizations can be made to illustrate how the neural network perceives the vocalizations, and therefore produce maps of the observed vocabulary (Fig. 2).

The t-SNE mapping of the valence-trained neural network (Fig. 2a) exhibits strong, but not complete differentiation between positive and negative vocalizations. The neighborhoods that exhibit extensive mixing indicate a hazy boundary between positive and negative calls. In the clusters where the vast majority of points are of a single valence, the presence of several irregular points demonstrates outlier vocalizations in the dataset, which might be calls for which the valence was incorrectly assumed.

The t-SNE mapping of the context-trained neural network (Fig. 2b) shows remarkably clear clusters, despite the large range in the number of vocalizations per context class (e.g. Surprise: 17, Isolation: 2069). However, the smaller classes have generally less clear boundaries, likely due to the neural network’s lower incentive to recognize them during training because of the class imbalance^23^. Notably, several of the larger context categories have split into two or more clusters (like Reunion, and arguably Isolation). In these cases, the network appears to be discerning subtypes within the context categories beyond what it was trained to recognize. These distinctions are due to the composite nature of the dataset; for instance, ‘Reunion’ experiments were conducted by two different teams. It is therefore unclear whether these experiments, using slightly different protocols, produced markedly different vocalization types, or if the environmental noise captured by the recording teams causes this subdivision. Inversely, it can be seen that some contexts that were expected to be distinct produced indiscernible calls (e.g. negative and positive conditioning; Fig. 2b). However, further analyses suggest that the environmental noise likely did not affect the valence and context classification (see Supplementary Text, Supplementary Figure S5, and Supplementary Tables S4-S5 for further information on this analysis).

## Discussion

Over the past 15 years, the interest in vocalizations as candidates for developing real-time, automated monitoring of animal emotions and welfare on-farm has considerably increased^9,16,24^. However, most experimental attempts have focused on just a few contexts and a limited age range. Here, we gathered recordings from five research laboratories with expertise on pig vocalizations to include 19 context categories covering the whole life of commercial pigs (411 pigs in total). Despite variability in age, sex, body size, and situation, we showed that the assumed emotional valence (for LF calls) and the context of vocal production (for both LF and HF calls) can be correctly cross-classified above chance levels from a small number of selected vocal parameters (pDFA). By using a neural network to classify spectrograms of the entire vocalizations, classification accuracy can be greatly increased. These results suggest that an automated recognition system can be developed for this highly commercial species to allow real-time discrimination of emotional states by valence or context of production. To our knowledge, none of the currently existing monitoring technology (Precision Livestock Farming) developed for pigs can assess the valence of the animals’ emotions^25^. Such a system would thus be highly useful to enable famers to keep track of this important component of animal welfare.

### Effect of valence and context on specific vocal parameters

Our results show that the acoustic structure of both LF and HF calls vary according to the emotional valence (negative vs. positive) and the context of vocal production (19 contexts). Two of the acoustic parameters, the duration (Dur) and amplitude modulation rate (AmpModRate), decreased from negative to positive valence for both call types. This suggests that positive calls, whether they are LF or HF, are shorter and contain less amplitude modulations than negative calls. In particular, measures of *R*^2^ indicated that 27% of the variance in the duration LF calls, and 30% of the variance in the duration HF calls, was explained by the emotional valence alone, which can be interpreted as large effects (*R*^*2*^ > 0.25 ^26^). By contrast, for the other parameters measured in LF and HF calls (spectral center of gravity (Q50%) and Wiener Entropy (WienEntropy)) only 1% to 10% of the total variance was explained by the emotional valence alone. The observation that shorter vocalizations are associated with positive emotions corroborates previous finding in domestic pigs^17,18,20,21,27^, as well as wild boars^28^. This association appears to be a common pattern among the species in which the effect of valence on vocalizations has been studied so far^10,11^. In addition, this pattern does not seem to be due to a confounding effect of emotional arousal, which could result from positive contexts included in our analyses being associated with an overall lower emotional arousal compared to negative contexts, since it is observed also in studies in which arousal has been controlled (e.g.^20,28^, or at least is expected to be similar^21^). It should be noted that Dur tends to increase with emotional arousal in some species, but often also shows the opposite pattern^11^. The decrease of AmpModRate from negative to positive valence also corroborates previous studies in wild boars^28^ and Przewalski’s horses^29^ suggesting a universality of the encoding of emotions in vocalization. Changes in Dur and AmpModRate are thus good candidates for further development of automated systems aimed at recognizing emotional valence, although this would require a system that includes an automated call detection to identify call onset and offset in noisy farming environments.

Interestingly, the two other parameters included in our analyses, Q50% and WienEntropy, showed opposite patterns in LF and HF calls. Indeed, Q50% increased from negative to positive contexts in LF calls, while it decreased in HF calls. WienEntropy showed the opposite pattern. Such specific patterns of change in vocal parameters with emotions has also been found in relation to arousal in pigs^19^, and in relation to valence in wild boars^28^ and Przewalski’s horses^29^. Those patterns could be due to differences in the vocal production mechanisms underlying these various call types, or in their function. An increase in energy distribution (Q25%, Q50% or 75%) between negative and positive contexts in LF calls is consistent with previous findings in low, closed mouth grunts (LF^18,21^) and in barks (also LF^30^), and could constitute another good candidate for the development of a system that could automatically recognize valence. This would, however, require the implementation of a first step, during which a distinction between LF and HF calls is made based on the spectral center of gravity (Q50%).

The pattern found for WienEntropy, which assesses the noisiness of a vocalization is less clear, as LF calls were more noisy (less tonal or ‘periodic’), while HF calls were less noisy (more tonal), in negative compared to positive contexts. This is in contrast with recent results, showing that LF calls (e.g. grunts) are less noisy (higher harmonicity) in a negative compared to a positive situation of similar arousal level^20^. Harmonicity has also previously been shown to decrease (indicating more noisy calls) in LF (grunts) and increase (indicating less noisy calls) in HF (screams) with emotional arousal^19^. The results we found might thus be explained by some of the negative contexts (e.g., particularly castration and slaughterhouse recordings) being strongly invasive and nociceptive, which could have induced emotions of higher arousal compared to the positive contexts. Hence, WienEntropy might not be a consistent candidate to include in an automated system for valence recognition, due to its sensitivity to changes in emotional arousal (confounding effect).

Regarding the effect of the context, the vocal parameters tested in our analyses (Dur, AmpModRate, Q50% and WienEntropy) all varied with the characteristics of the context in which calls were produced. Changes to the various parameters were largely in accordance with the changes due to emotional valence that we describe above, suggesting that context-related changes might be primarily due to their valence.

### Automated classification

#### Permuted discriminant function analysis

Through a two-step procedure including first the distinction between LF and HF calls and then a discrimination based on the four acoustic parameters explaining most of the variance in the data, both the valence (for LF calls) of the contexts and the actual contexts of production (for both LF and HF calls) could be correctly cross-classified above chance levels. For the valence, the classification of calls used for deriving the discriminant functions (i.e. no cross-classification) reached a rather high success of above 80% for the LF calls and 95% for the HF calls. However, when using a more conservative approach and classifying calls not used for deriving the discriminant functions (cross-classification), the percentage of calls attributed to the correct valence dropped to 61% for LF and 63% for HF. In addition, the percentage of correctly attributed HF calls was not significantly higher than chance, likely due to the low prevalence of HF calls in positive (*n* = 225 calls) compared to negative (*n* = 1676 calls) contexts (Supplementary Table S2). Yet, these results indicate that a system based on a few acoustic parameters is capable of correctly detecting in some cases, from a single call, whether a pig is in a positive or a negative situation. The results are in agreement with Tallet et al.^17^, who found that classification into three gross biological types of contexts (life threat/nursing/other) could be accomplished with a success rate of 75% for a single call on the basis of eight acoustic variables. The potential classification success of an automated device could be further improved if it would use for the valence assessment not just a single call, but a number of calls. This is realistic as pigs commonly emit series of vocalizations. Using such an approach, an evaluation of about 10 calls may give a discrimination success that approaches 100% for a simple classification of emotional valence^17^.

For the classification of the actual context, the success was above chance, although many calls were misclassified, which is not surprising given the high number of different contexts (*n* = 19). In real farm situations, the number of possible contexts could be restricted by the set age/sex category of the pigs and the specific husbandry conditions/procedures. Such discrimination between only a few contexts would probably achieve a high success, even with a single call as previously documented for a 3-context case^17^. Additionally, the principle of using more calls may also be applied to the assessment of the context. Conceivably, an on-farm system using multiple calls and tailored to a specific category of pigs, and thus limited to a low number of possible contexts, could aspire to a much higher level of discrimination.

#### Neural network

The spectrogram classifying neural network appears extremely promising, due to its high accuracy and minimal audio pre-processing. As the frequency of a vocalization is encoded within its spectrogram, the method merely needs an audio file cropped to the length of the vocalization, without first discerning if it is LF or HF, which requires the age of the vocalizer to be known. The process of appropriately cropping an audio file could also be fully automated by using for instance region based CNN^31^, and therefore, this method could be readily implemented towards a real-time classification tool. The achieved accuracy by the neural network method for valence classification (91.5%) is much higher than that of the pDFA analysis (weighted average across LF and HF of 61.7%). It should also be noted that the trained neural network is capable of classifying more than 50 spectrograms per second using the hardware of current smartphones, and does not require the extraction of vocal parameters that is needed for the pDFA, so this should not present an obstacle. With regard to context classification accuracy, the neural network performs, again, much more strongly than the pDFA analysis (81.5% vs. weighted average across LF and HF of 19.5%). This is largely to be expected, as using four parameters to predict 18 categories is highly difficult. In this case, a neural network that analyses spectrograms of entire vocalizations is able to preserve more encoded information, and can thus make much stronger predictions. Though the neural network performs well here, it could likely be improved by as much as 10% by addressing the imbalance in context classes^23^.

To conclude, in this study, we collaboratively built a large database of vocalizations spanning the lives of pigs from birth to slaughter, analyzed it for acoustic insights, and tested two potential classification methods. First, the acoustic analyses revealed that emotional valence can be inferred by call duration and amplitude modulation rate. The spectral center of gravity (Q50%) seems to be an additional promising indicator for increasing the accuracy of an automated system for recognizing emotional valence in calls. Second, using just a small number of acoustic parameters, we found that the emotional valence (for LF calls) and context of production of vocalizations (for both LF and HF calls) could be cross-classified above chance levels (61.7% for valence with a 50.5% chance level; 19.5% for context with a 14.3% chance level) using a pDFA analysis. The second classification approach, a spectrogram classifying neural network, classified vocalizations with a much higher accuracy by valence (91.5%) and context (81.5%). In combination with t-SNE, this method could be used to refine the dataset, identify novel vocalization types and subtypes, and further expand the recognizable vocabulary of animal vocalization. The classification successes achieved in this study are encouraging to the future development of a fully automated vocalization recognition system for both the valence and context in which pig calls are produced. Such system should then ideally be externally validated, and its performance assessed, in order to establish its potential for a wide and useful implementation. Considering the high accuracy (≥ 81.5%) reached by the neural network in our study, we believe that the performance of this system could be similar, or higher, than the performance of existing microphone-based systems, which are aimed at classify stress vocalizations and coughing (> 73%^25^).

## Methods

### Recording contexts

In order to consider situations typically encountered by commercial pigs throughout their life, we first gathered vocalizations that had been recorded as part of previously published studies (Supplementary Table S1), and completed our database with recordings collected for the specific purpose of the current analysis. The final database consisted of over 38,000 calls recorded by five research groups, representing 19 context categories (see Supplementary Table S1 for information on the number of calls, animals, their age, breed, and sex across the contexts).

### Determination of the valence of contexts

The valence of the contexts was determined based on intuitive inference, within the two-dimensional conceptual framework^4,32^. Negative emotions are part of an animal’s unpleasant-motivational system and are thus triggered by contexts that would decrease fitness in natural life and are avoided by pigs; such contexts (e.g., stress, social isolation, fights, physical restraint) were thus assumed to be negative (Supplementary Table S1). Similarly, positive emotions are part of the pleasant-motivational systems and occur in situations contributing to increased fitness. Such situations (e.g., reunion, huddling, nursing, positive conditioning), which trigger approach or search behavior in domestic pigs were thus assumed to be positive (Supplementary Table S1)^4,33^.

### Acoustic analyses

In total, 7414 calls were selected from the database based on their low audible/visible (in the spectrogram) noise (i.e. low signal-to-noise ratio that distorts acoustic characteristics of the calls or impedes the precise detection of call onset and end; see Supplementary Text for further details on this selection), and analyzed using a custom-built script in Praat v.5.3.41 DSP Package^34^. This script batch processed the vocalizations, analyzed the parameters and exported those data for further evaluation (adapted from^20,35–37^). In total, we extracted 10 acoustic parameters that could be measured in all types of calls and were likely to be affected by emotions (Table 3; see Supplementary Text for detailed settings^11,17,18,38^). Calls were classified into two types, i.e., low-frequency calls (LF) or high-frequency calls (HF) based on their extracted spectral center of gravity (Q50%) (cut-off point between LF and HF: age class 1 (1–25 days old) = 2414 Hz; age class 2 (32–43 days old) = 2153 Hz and age class 3 (≥ 85 days old) = 896 Hz; See Supplementary Text for further details). Overall, our analyses included 2060 positive LF calls, 3453 negative LF calls, 225 positive HF calls, and 1676 negative HF calls (Supplementary Table S1 and S2).

**Table 3:** Acoustic parameters.

Abbreviation	Description	Category	Reference
Dur (s)	Duration of the call	Duration	^17–21,27,30,39–41^
AmpVar (dB/s)	Amplitude variation; cumulative variation in amplitude divided by the total call duration	Amplitude modulation	^20^
AmpModRate (s-1)	Amplitude modulation rate; number of complete cycles of amplitude modulation per second		^21,28^
AmpModExtent (dB)	Amplitude modulation extent; mean peak-to-peak variation of each amplitude modulation		^21,28^
Q25% (Hz)	Frequency value at the upper limit of the first quartiles of energy	Spectrum (energy distribution)	^18,20,21,27,28,30,39,40^
Q50% (Hz)	Spectral center of gravity; frequency value at the upper limit of the second quartiles of energy		^17–21,27,28,30,39,40^
Q75% (Hz)	Frequency value at the upper limit of the third quartiles of energy		^18,20,21,27,28,30,39,40^
FPeak (Hz)	Frequency of peak amplitude		^17,18,20,30,39,41^
Harmonicity	Degree of acoustic periodicity, also called harmonic-to-noise ratio—higher values indicate more tonal calls	Tonality/noise	^18–21,28,30,39,40^
WienEntropy	Wiener entropy; spectral flatness of a sound, calculated as the ratio of a power spectrum's geometric mean to its arithmetic mean measured on a logarithmic scale—higher values indicate more noisy calls		^17,18,27,39,41^

Abbreviation and description of the analyzed acoustic parameters, along with the category they were allocated to, which was used to select the best parameters to include in our analyses, as well as examples of references to other studies where these parameters were measured in relation to emotions in pig and wild boars.

### Statistical analyses

#### Changes to specific vocal parameters

Since our 10 acoustic parameters were likely to be inter-correlated, we first carried out a principal component analysis (PCA) in R software v.3.6.1. (prcomp function, package stats^42^) on each call type (LF and HF) separately (two PCAs in total), in order to select a set of non-redundant parameters. This procedure resulted in the following four parameters to be included in subsequent tests: Dur, AmpModRate, Q50% and WienEntropy (Supplementary Table S3; see Supplementary Text for more information on the PCA).

To investigate the effect of the assumed valence (positive or negative) and of the context category (19 categories; Supplementary Table S1) on the acoustic structure of the calls, the raw values of these selected parameters (Dur, AmpModRate, Q50% and WienEntropy) were entered as outcome variables into linear mixed-effects models (LMM; one model per outcome variable) fit with Gaussian family distribution and identity link function in R software v.3.6.1. (lmer function, package lme4^43^). Since the effects of emotions on acoustic parameters are likely to vary between call types (in pigs^19^ and also in other species, including wild boars^28,29^), and since the variance in each parameter differs between calls types, LF and HF calls were analyzed separately. These models included either the assumed valence of the situation (positive or negative; 8 models in total) or the context category (19 categories; Supplementary Table S1; 8 models in total) as fixed factors. In addition, the age class (3 classes; 1 = 1–25 days old, 2 = 32–43 days old, 3 ≥ 85 days old) was included as a fixed factor to control for its effect on the acoustic structure of the calls. Our models included the identity of the pigs (*n* = 411 pigs), nested within the team who provided the recordings as a random effect (Supplementary Table S1), to control for dependencies between values collected on the same pigs and by the same team. Only the results of the fixed factors of interest (valence and context category) are described in the results (see Supplementary Text for more information on the LMMs).

#### Automated classification

##### Permuted discriminant function analysis

To test if calls could be classified to the correct valence or context category above chance levels, further analyses were carried out on the same selected four parameters (Dur, AmpModRate, Q50% and WienEntropy). This was achieved using a permuted discriminant function analysis (pDFA^44^), which can handle unbalanced datasets and allows the inclusion of a control factor. The pDFA was conducted using a script provided by R. Mundry, based on the function lda of the R package MASS^45^ (see Supplementary Text for more information on the pDFA settings). Since not all pigs were recorded in both valences, nor in all context categories, we used a crossed pDFA for incomplete design. It included either the valence (positive or negative) or the context category (19 categories; Supplementary Table S1) as the test factor, and the individual identity of the pigs (*n* = 411 pigs) as the control factor.

##### Neural network and t-SNE

To complement and contrast the pDFA classifier, a second approach which preserved as much signal information as possible was desired. For this task, a machine learning algorithm was chosen as they are optimal for analyzing complex, high-dimensional data. A neural network was selected because of the minimal pre-processing and data reduction required. Additionally, neural networks have proven highly capable in sound classification tasks^46–48^. The convolutional neural network ResNet-50 was chosen to be adapted via transfer learning because of its performance efficiency^49^ and proven application in this field^47^. As an input to the neural network, spectrograms were computed from the pig vocalization audio recordings in MATLAB R2020b. Each spectrogram was centrally zero-padded to be of equal length to the longest recording (3.595 s). The spectrograms were computed using a 3 ms window, 99% overlap, and 512 sampling points to calculate the Discrete Fourier Transform. Neural networks were trained separately to both desired applications: (1) classifying the spectrograms based on positive or negative valence; and (2) classifying the spectrogram according to the context in which the vocalization was produced.

The dataset was randomly split 70/30 into a training and validation set each time the neural network was trained. The neural network was trained on the given classification task (valence/context) for 20 epochs, with a mini-batch size of 32, an initial learning rate of 0.001, and a learn rate drop factor of 10^–0.5^. After the training period, the highest accuracy version of the neural network, as measured on the validation set, was saved. This was repeated 10 times for each classification task, in order to assess the consistent performance ability of the neural networks (Table 2) (see Supplementary Text for more details on the neural network and t-SNE analysis and validation).

### Ethics declarations

All experiments were performed in accordance with relevant guidelines and regulations and approved by the respective authorities for each country (Germany: Federal State of Mecklenburg-Western Pomerania (AZ:7221.3-2-045/13); Switzerland: Swiss Cantonal Veterinary Office (TG02/2014); Czechia: Institutional Animal Care and Use Committee of the Institute of Animal Science and the Czech Central Committee for Protection of Animals, Ministry of Agriculture (dMZe 1244 and 44248/2007-17210); Norway: National animal research authority (FOTS id 12021)). The reporting in the manuscript (see Supplementary Text and Supplementary Table S1) follows the recommendations in the ARRIVE guidelines.

### Supplementary Information

Supplementary Information 1.

Supplementary Information 2.

## Data Availability

The raw data are included as a supplementary file (Dataset S1). The database of labelled pig call audio recordings is available at: https://zenodo.org/record/8252482.
