# Author Correction: Classification of pig calls produced from birth to slaughter according to their emotional valence and context of production

**DOI:** 10.1038/s41598-023-45242-9

**Published:** 2023-11-01

**Authors:** Elodie F. Briefer, Ciara C.‑R. Sypherd, Pavel Linhart, Lisette M. C. Leliveld, Monica Padilla de la Torre, Eva R. Read, Carole Guérin, Véronique Deiss, Chloé Monestier, Jeppe H. Rasmussen, Marek Špinka, Sandra Düpjan, Alain Boissy, Andrew M. Janczak, Edna Hillmann, Céline Tallet

**Affiliations:** 1 Institute of Agricultural Sciences, ETH Zurich, Universitätsstrasse 2, 8092 Zürich, Switzerland; 2 Behavioural Ecology Group, Section for Ecology and Evolution, Department of Biology, University of Copenhagen, 2100 Copenhagen, Denmark; 3 Department of Ethology, Institute of Animal Science, 104 01 Prague, Czechia; 4 Department of Zoology, Faculty of Science, University of South Bohemia, 370 05 Č. Budějovice, Czechia; 5 Institute of Behavioural Physiology, Research Institute for Farm Animal Biology (FBN), 18196 Dummerstorf, Germany; 6 Department of Agricultural and Environmental Sciences, Università Degli Studi Di Milano, Milano, Italy; 7 Faculty of Veterinary Medicine, Norwegian University of Life Sciences, Universitetstunet 3, 1433 Ås, Norway; 8 PEGASE, INRAE, Institut Agro, 35590 Saint Gilles, France; 9 University of Clermont Auvergne, INRAE, VetAgro Sup, UMR Herbivores, 63122 Saint‑Genès Champanelle, France; 10 Bureau ETRE, 63210 Bravant, Olby France; 11 Center for Coastal Research, University of Agder, 4604 Kristiansand, Norway; 12 Center for Artificial Intelligence Research, University of Agder, 4604 Kristiansand, Norway; 13 Faculty of Agrobiology, Food and Natural Resources, Czech University of Life Sciences, 165 21 Prague, Czechia; 14 Animal Husbandry and Ethology, Albrecht Daniel Thaer‑Institut, Faculty of Life Sciences, Humboldt-Universität Zu Berlin, Philippstrasse 13, 10115 Berlin, Germany; 15 School of Engineering and Applied Sciences, Harvard University, Cambridge, MA USA

Correction to: *Scientific Reports* 10.1038/s41598-022-07174-8, published online 7 March 2023

The Data availability section in the original version of this Article was modified.

“The raw data are included as a supplementary file (Dataset S1).”

now reads:

“The raw data are included as a supplementary file (Dataset S1). The database of labelled pig call audio recordings is available at: https://zenodo.org/record/8252482.”

The original Article has been corrected.

