# Supplementary material for: Classification of pig calls produced from birth to slaughter according to their emotional valence and context of production
Source: Sci Rep. 2022 Mar 7;12:3409. doi: 10.1038/s41598-022-07174-8 (PMC8901661; doi:10.1038/s41598-022-07174-8)
Supplement: Supplementary file 1 — Supplementary Information 1. [file 41598_2022_7174_MOESM1_ESM.docx]

**Supplementary Information for**

**Classification of pig calls produced from birth to slaughter according to their emotional valence and context of production**

Elodie F. Briefer^1,2*^, Ciara C.-R. Sypherd^2,3^, Pavel Linhart^4,5^, Lisette M.C. Leliveld^6,7^, Monica Padilla de la Torre^8^, Eva R. Read^9^, Carole Guérin^9^, Véronique Deiss^10^, Chloé Monestier^11^, Jeppe H. Rasmussen^6,12,13^, Marek Špinka^4,14^, Sandra Düpjan^6^, Alain Boissy^10^, Andrew M. Janczak^8^, Edna Hillmann^1,15^, Céline Tallet^9^

^1^ Institute of Agricultural Sciences, ETH Zurich, Universitätsstrasse 2, 8092 Zürich, Switzerland

^2^ Behavioural Ecology Group, Section for Ecology & Evolution, Department of Biology, University of Copenhagen, 2100 Copenhagen Ø, Denmark

^3^ School of Engineering and Applied Sciences, Harvard University, USA

^4^ Department of Ethology, Institute of Animal Science, 104 01 Prague, Czechia

^5^ Department of Zoology, Faculty of Science, University of South Bohemia, 370 05, Č. Budějovice, Czechia

^6^ Institute of Behavioural Physiology, Research Institute for Farm Animal Biology (FBN), 18196 Dummerstorf, Germany

^7^ Department of Agricultural and Environmental Sciences, Università degli Studi di Milano, Milano, Italy

^8^ Faculty of Veterinary Medicine, Norwegian University of Life Sciences, Universitetstunet 3, 1433 Ås

^9^ PEGASE, INRAE, Institut Agro, 35590 Saint Gilles, France

^10^ University of Clermont Auvergne, INRAE, VetAgro Sup, UMR Herbivores, F-63122, Saint-Genès Champanelle, France

^11^ Bureau ETRE, Bravant, 63210 Olby, France.

^12^ Center for Coastal Research, University of Agder, 4604, Kristiansand, Norway

^13^ Center for Artificial Intelligence Research, University of Agder, 4604 Kristiansand, Norway

^14^ Faculty of Agrobiology, Food and Natural Resources, Czech University of Life Sciences, 165 21 Prague, Czechia

^15^ Animal Husbandry and Ethology, Albrecht Daniel Thaer-Institut, Faculty of Life Sciences, Humboldt-Universität zu Berlin, Philippstrasse 13, 10115 Berlin, Germany

* **Corresponding author:** EFB, elodie.briefer@bio.ku.dk

**This PDF file includes:**

Supplementary Text

Supplementary Figures S1 to S5

Supplementary Tables S1 to S5

Supplementary References

**Other supplementary materials for this manuscript include the following:**

Dataset S1

Supplementary Text

**Description of contexts not appearing in published articles**

**Additional experiments conducted by NMBU**

***Subjects and study site.*** Two experiments were conducted using the same subjects: a conditioning experiment, during which pigs were conditioned to expect an enriched or a barren environment, and a running test, during which pigs were let free to run in a corridor. Both experiments were conducted at the pig research facility of the Norwegian University of Life Sciences (NMBU) in Ås, Norway, from May to June 2017. The animals used for both experiments (n=16 female and 16 male pigs, Noroc breed (TDLZL; crossbreed Tn70 x Duroc)) were randomly selected from four different litters. A total of 16 sibling pig pairs were housed on solid floored pens (1.0 m wide × 2.6 m deep) with visual, auditory and olfactory contact with conspecifics. The pens were covered with a layer of sawdust, which was changed every morning. Pig pairs were given *ad libitum* access to water and were fed according to swine industrial standards. The 16 pairs of pigs experienced 5 weeks of habituation (conditioning) that consisted of herding each pair of pigs though a long corridor (2.08 m wide m x 23.33 m long) from the home pen to an experimental arena (either barren or enriched) where they were kept for 10 minutes. The barren arena (3.0 m wide x 2.80 m long; 8.4 m^2^) had a solid concrete floor and was totally empty. The enriched arena (3.0 m wide x 2.90 m long; 8.7 m^2^) also had a solid concrete floor but was covered with a thick layer of straw. Additionally, plastic toys and food treats were scattered around the whole arena. The toys were a rubber pig, a rubber horseshoe, a yarn braid toy and cardboard boxes. The food treats were: freshly sliced apples (two apples per trial), corn flakes (approximately 100 g), puffed rice bread in pieces (approximately 100 g), pasta (approximately 100 g) and 100-200 g of silage.

***Conditioning experiment.*** All 16 pairs of pigs experienced both the barren and enriched arenas once per week during a total of 7 weeks (5 weeks of habituation, and 2 weeks of testing, during which video and audio recordings were collected), on alternating days as follows; on the first day of each week, for the pig pairs 1-8, the odd numbers (i.e., pairs 1, 3, 5 and 7) were taken to the enriched arena while even pair numbers (i.e., pairs 2, 4, 6 and 8) were taken to barren arena. Pig pairs 9-16 remained in their home pens during that day. The following day, for pig pairs 9-16, the odd numbers (pairs 9, 11, 13 and 15) were taken to enriched arena, while even pair numbers (pairs 10, 12, 14 and 16) were taken to the barren arena. Pig pairs 1-8 remained in their home pens during that day. The following two days of the week, the same procedure was repeated but alternating the arenas between pairs. During the two last weeks, pig vocalizations were recorded in each test trial using a Sennheiser MKH70 directional microphone, connected to a Marantz PMD660 digital recorder (sampling rate 44.1 kHz, 16 bit, mono, wav format). The microphone and digital recorder were mounted on a tripod that was standing next to the fence of each arena (either barren or enriched) and remained out of sight of the pigs (1.20 m, above the fence). Pig behavior was video recorded using a Panasonic analog camera (WV-CP500/G) that was suspended from the ceiling, positioned so that it faced the middle of the test arena and connected to a computer with EthoVision XT 10 (Noldus Information Technology, Wageningen, The Netherlands).

***Running experiment.*** After 7 weeks following the same treatment (see above), pigs were recorded while running in the corridor over 4 days. Each running trial consisted in one pair of pigs (pairs 1-16) being released from their home pen (opening a metallic gate) and set free to run towards the experimental arena. Once they reached the experimental arena, the pigs were left in either a barren or an enriched arena (as descried above) for 5 minutes. Afterwards, the pair of pigs were set free to run back from the experimental arena towards the house pen. Therefore, overall, a total of 4 running trials (both ways) were recorded from each pair of pigs. The equipment was set up above the one fence when the pigs were running towards the experimental arena, while on the way back (from experimental arena towards house pen), the equipment was placed in a corner on a tripod. The audio and video recordings were exclusively made when the pigs were on running in the corridor. Each trial was recorded in in the same format and with the same equipment as for the conditioning experiment, and filmed using a digital video camera (Sony DSC-HX300).

The Norwegian Food Safety Authority that approved all the experimental procedures carried out with the pigs (Application, FOTS ID 12021)

**Additional experiments conducted by IASP**

The facilities and subjects are described in details in Linhart et al.^1^. The piglets went through a battery of several behavioral tests and simulated situations including back-test (see Linhart et al.^1^ for details), isolation, reunion, and real and sham castration. Seven day-old piglets were individually taken out of the pen and transported to another room with no acoustic contact with other pigs. Here, the piglet was placed inside a small box (0.7m x 0.7m x0.7m) opened from the top and subjected to a short (3 minutes) or long (8 minutes) isolation. Their vocalizations were recorded with a directional microphone set up 1 meter above the box (Sennheiser ME66) and digital recorder (Marantz PMD671, sampling rate: 48 kHz, 24 bit, mono, uncompressed PCM format). Immediately after the isolation, piglets were transported back to their home pen and their immediate vocal response to reunion was recorded for 1 minute. Piglets were further recorded during the routine castration procedure on the farm. Castration was done by an experienced stockperson and no anaesthetics were used for the purpose of the experiment. Recordings were collected at 2 meters from the subject in a separate room, using the same equipment as for the isolation and reunion situation. Half of the piglets went immediately through the castration procedure while the second half of the piglets went through sham castration first. During sham castration, a piglet was handled, fixed and treated like in a real castration, but incisions were only simulated by the blunt side of scalpel. The stage of the procedure was noted on the microphone.

This experiment was approved by the Institutional Animal Care and Use Committee of the Institute of Animal Science and the Czech Central Committee for Protection of Animals, Ministry of Agriculture (decision MZe 1244).

**Additional experiments conducted by INRAE and ETRE**

Pig sounds were recorded in a French commercial slaughterhouse during routine slaughter. Pigs were transported in groups to the slaughterhouse the day before their slaughter and were submitted to a period of lairage during approximately 12 hours during which they were food deprived. They were then introduced into an individual slaughter corridor, using hand/voice or electric prod, and remained without human presence during a few minutes. They were introduced into the restraining device, a V-Type restrainer conveyor. It holds the animal between two conveyor belts, set in a ‘V’ formation. The speed of the restrainer-conveyor can be varied, according to the type of animal and capabilities of the individual operator. The system is usually operated with a foot pedal. At the end of the restrainer conveyor, pigs were individually stunned using an electrical stunning.

Recording of pig sounds were carried out during 4 different stages, all corresponding to an unfamiliar and noisy environment: 1) Waiting in the individual slaughter corridor near the slaughter area; 2) Handling in the individual slaughter corridor by an unfamiliar human using voice and/or hand to handle pigs; 3) Handling in the individual slaughter corridor by an unfamiliar human using an electric prod^2^; 4) Introduction of pigs into the restrainer conveyor.

Recordings were carried out during 3 slaughter days, May 10, April 19 and 26 between 6:00: and 10:00 am, using a microphone (Sennheiser ME 67), connected to a recorder (Marantz PMD661; sampling rate: 44.1 kHz, 16 bit, mono, wav format).

**Acoustic analyses**

**Initial selection of calls**

Due to the large amount of calls in our original database (n > 38000 calls), an initial selection was carried out as follows; we used all the sounds available for all situations, except for two recording situations that were highly overrepresented: ‘Social isolation in a small box for 3 min’ and ‘Social isolation in a small box for 8 min’ (see Supplementary Table S1 for details), with 29196 individual calls. For these two contexts, we selected a random representative sample of about 1000 calls, equally divided between males and females, and between the two types of isolation (3 or 8 minutes). During the acoustic analysis, we also manually excluded calls whose quality was not estimated to be good enough for further analyses, i.e. with a sound to background noise ratio that was too low (as assessed visually on a spectrogram).

**Setting**

We provide here a more detailed description of the acoustic analysis. The settings entered in the script used to extract the 10 acoustic parameters from low- and high-frequency (LF and HF) calls are detailed below in the order presented in Table 2 (Praat commands are indicated in brackets; see Table 2 for abbreviation of the parameters).

1. Duration. The duration (Dur) was measured as the total duration of each wav file, which corresponded to individual calls previously extracted manually from the recordings, based on the visualization of both the oscillogram and spectrogram.
2. Amplitude modulation. AmpVar, AmpModRate and AmpModExtent were calculated from the intensity contour of each call extracted using the [Sound: To Intensity] command (Minimum pitch = 150 Hz, Time step = 0.008 s), with the method described in Charlton et al.^3^. The minimum pitch was set as such so that short calls (but not shorter than 0.043 s, corresponding to 6.4/150 Hz) could be processed.
3. Spectrum-related parameters. Q25%, Q50% and Q75% were measured on a spectrum applied to the whole call, and FPeak was measured on a cepstral-smoothed spectrum ([Create: Cepstral smoothing] command; Bandwidth = 100 Hz).
4. Noise. We measured the Harmonicity using the [Sound: To Harmonicity (cc)] command (time step = 0.008 s, minimum pitch = 150 Hz, Silence threshold = 0.2, Periods per window = 1), and the Wiener entropy (WienEntropy) using a script provided by Beckers^4^ with the following settings; frame duration = 0.01 s, time step = 0.004 s, start frequency = 50 Hz, end frequency = Q75%.

AmpModRate and AmpModExtent could not be extracted from 191 calls. All other parameters could be measured in all calls.

**Classification into low and high frequency calls**

Since visual classification of calls can be biased, we established a cut-off point to separate LF from HF calls. Calls were initially a-priori classified based on their acoustic structure, visualized on spectrograms (FFT method, window length = 0.01 s, time steps = 1000, frequency steps = 250, Gaussian window shape, dynamic range = 60 dB, view range = 0-8000 Hz), into five main call types according to Tallet et al.^5^, as follows; low-frequency stable (LFs, e.g. closed-mouth grunt), low-frequency modulated (LFm, e.g. open-mouth grunt), low-frequency tonal (LFt, e.g. bark, croak or chirrup), high-frequency stable (HFs, e.g. squeal or squeak), and high-frequency modulated (HFm, e.g. scream). In addition, any call consisting of a mix between two of these categories was labelled as ‘mixed’ (e.g. grunt-scream; total = 6 call types). To ensure reproducibility, this classification was performed for each context by two trained people. Following this a-priori classification, a discriminant function was carried out in R software (v.3.6.1) to identify which acoustic parameter varied the most between LF calls (including LFs, LFm and LFt) and HF calls (including HFs and HFm). The spectral center of gravity (Q50%) was the parameter that obtained the highest loading on the first discriminant function (LD1; r = -0.95) and was thus chosen for establishing a cut-off point. The cut-off point was then determined for the 3 age classes of pigs separately (1 = 1-25 days old, 2 = 32-43 days old, 3 ≥ 85 days old) as follows; since the number of calls differed between call types and age categories, for each, we calculated the average median of 1000 random selections of 100 Q50% values. For a given age class, the cut-off point was then determined as the difference in the resulting median for HF and LF calls (class 1 = 2414 Hz; class 2 = 2153 Hz and class 3 = 896 Hz). These cut-off points resulted in the classification of 88.7% (age class 1) to 99.8% (age class 3) a-priori LF calls (LFS, LFm and LFt) as LF, and in the classification of 82.7% (age class 3) to 86.5% (age class 1) a-priori HF calls (HFs and HFm) as HF. Regarding mixed calls, 38.4% (age class 2) to 58.4% (age class 3) were classified as HF. All statistical analyses were conducted on LF and HF calls classified based on these cut-off points.

**Statistical analyses**

**Principal component analysis (PCA)**

The principal components (PC) with an eigenvalue greater than 1 (Kaiser’s criterion: 4 in LF and 3 in HF calls; Supplementary Table S3) were extracted from the PCA and the parameters loading highly (r >= l0.5l) on each PC were highlighted. Then, for each category of parameters (see Table 1 for details), we selected the parameter that had the highest loading on each PC. Finally, when the category contained several parameters, the parameter kept for further analyses was the one being selected the most often across PCs and call types.

**Linear mixed-effects models (LMM)**

We checked the residuals of the models graphically for normal distribution and homoscedasticity (simulateResiduals function, package DHARMa^6^). To fit the model assumptions, Dur and AmpModRate were log transformed. P values (PBmodcomp function, package pbkrtest^7^) were calculated using parametric bootstrap methods (1000 samples). Model estimates and confidence intervals were calculated for all models using a bootstrap approach (1000 samples, bootMer function, package lme4^8^). In addition, we calculated marginal *R*^2^ (*R^2^*_GLMM(m)_) of our fixed factor (valence or context category) following^9^. *R^2^*_GLMM(m)_ corresponds to the proportion of variance explained by the fixed factor alone.

**Permuted discriminant function analysis (pDFA)**

When testing the effect of the valence, it was possible to set the argument for balanced dataset to ‘mode’, since some pigs had been recorded in both positive and negative contexts. In this case, a repeated random selection of same number of cases per combination of test and control factor as well as of same number of levels of the test factor per level of the control factor is used for deriving the discriminant function(s). By contrast, when testing the effect of the context, since each pig had been exposed to only a few of each, we had to set the argument to ‘no’, resulting in all available combinations of the levels of the test factor and the control factor being chosen. Although the pDFA is rather robust against outliers or skewed distributions, Dur and Q50% were log transformed to achieve approximately symmetrical distributions. The p value of a pDFA is calculated as the proportion of permutations revealing a number of correctly classified objects at least as large as the original data^10^. It should be noted that the results of a crossed pDFA for incomplete design should be interpreted with caution (R. Mundry, personal communication).

**Neural network**

***Neural network appraisal: accuracy vs. loss function***

While training the neural networks, a strong decorrelation between the cross-entropy loss function and the accuracy of the classifier was observed as the neural network approached peak accuracy performance. This is often indicative of outliers or misclassifications in a dataset, which the neural network attempts to better fit at the expense of accuracy. Combing the extensive dataset for outliers fell beyond the scope of this work, so this concern was abated by saving the neural networks and their performance metrics from the epoch with the highest accuracy (as measured on the validation set), rather than the loss function minima. This more faithfully appraises the potential for neural networks as a classifier of pig vocalizations, but still likely provides an underestimate of the neural networks’ abilities.

***Visualizing the dataset and neural network performance***

The complex decision-making process of the proposed image classifying neural network begins with a spectrogram, and ends with a classification prediction. Along the way to making this classification, the neural network makes observations of key pixel arrangements, which are indicative of certain vocalization types. In the final layers of the neural network, these observations become a 2048-dimension vector representation of what the neural network deems to be the spectrogram’s most distinctive features, which finally allows the neural network to make a high-confidence classification. By examining the last fully connected layer of the neural network, it is possible to visualize how this intricate system perceived the dataset of vocalizations. This was done by applying a second machine learning algorithm, t-distributed Stochastic Neighbor Embedding (t-SNE), to the activations of the final fully connected layer produced by the neural network for each spectrogram.

t-SNE is a dimensionality reduction algorithm which faithfully recreates high-dimensional groupings in lower, visualizable dimensions. This is accomplished by calculating the probability that high-dimensional vectors are neighbors in their original high-dimensional space, and then faithfully recreating these probabilities as distances between points in lower dimensions ^11^. t-SNE has multiple tuneable hyperparameters, but only perplexity is of relevance here. Perplexity can be thought of as a measure of how many neighbors each point is expected to have ^11^. The selected perplexity values for Figure 2 are explained below.

For Figure 2a, a perplexity of 50 was chosen, as the valence groups are large (positive: 2285, negative: 5129) and so many neighbours are expected for each point. To avoid any confusion, it should be noted here that while t-SNE can be influenced by how many neighbours it is told to seek, it can only report relationships native to the original high-dimensional data. Therefore, it is interesting to explore the behavior of clusters at different perplexities, as this can inform about the relationships between vocalizations at various scales. It is thus of great interest that at perplexity 50 the t-SNE embedding groups the data into more than just two large neighbourhoods, as might be expected from the results of a binary classifier. The plurality of clusters here shows that the neural network detects more substructure in the dataset than just the two valences it was taught to identify. In fact, at lower perplexities, the given clusters further subdivide into smaller groups while maintaining the same global structure. These sub-clusters do not correspond well with context, recording team, sex, or age. These substructures are consequently expected to link to specific acoustic features observed in the spectrograms across the aforementioned categories.

Figure 2b was created by applying t-SNE to the context classifying neural network’s final fully connected layer activations for each spectrogram. Despite the large range in the number of vocalizations per context class (e.g. Surprise: 17, Isolation: 2069), the clusters in this map are robust to changes in perplexity across the tested range (10-50). As the smallest classes are naturally most distinct at low perplexities, a perplexity of 20 was chosen to be shown in this work.

***Neural network classifier verification***

A neural network was chosen for this classification task because neural networks operate on complex inputs, which allow for more complete, information rich signals to be analyzed. However, one drawback of this method can be the inclusion of undesired signal information, which may skew training. Specifically, since the dataset is a compilation of recording work done by multiple international collaborators, it was of great concern that the acoustic environment in which the vocalizations were recorded was not used by the neural network in order to aid in its classifications of the spectrograms. The term “acoustic environment,” here is meant to include differences in audio recordings caused by the team recording a vocalization, how far the microphone was from the pig, the use of particular recording equipment, the sounds of other pigs in the background, the sounds of farm equipment or machinery in the background, etc. For example, it can be seen in Figure 2b, there are two clearly distinct Reunion clusters, Restrain is split into multiple groups, and the Isolation neighborhood could arguably contain four hazily separated clusters. This could potentially be attributed to the fact that different teams, at separate recording locations, contributed these sound files. Perhaps the neural network and t-SNE are able to distinguish them, even within the same class label, by the environmental noise in the background. This argument can be supported by Figure S4, which shows the same data from Figure 2b, but colored according to which recording team contributed the data. However, it could also be true that these unexpected clusters are formed from distinctly different types of vocalizations, as no experiment was truly replicated in multiple environments. For example, while isolation experiments were conducted by three different teams at separate locations, the specifics of the experiment spanned placing a lone pig in a small pen, a small box, a familiar arena, and an unfamiliar arena. The period of isolation also varied between 3, 5, 8, and 10 minutes.

To quantitatively check how much the environmental noise of the site and recording team aided the neural network in making classifications, we trained a separate neural network to classify valence and context on only the vocalizations recorded by a given team. We then compared the weighted average of these networks’ “within recording team” context or valence classification accuracy to the “across recording teams” accuracies presented in Supplementary Table S3. We used the neural network trained across recording teams to try to predict the class of data from only one recording team at a time, for comparison. The resultant data is given in Tables S3 and S4. While the accuracies of the neural networks trained “within recording team” are higher than the accuracies of the network trained “across recording teams” which then predicts within recording team data, the variation of their accuracies is within a few percent, and the “within recording team” trained neural networks have the great benefit of being trained to classify less data, with fewer classes (in the case of context). While further statistical analysis should be undertaken to fully verify the role of environmental noise in this neural network’s classification of pig vocalizations, the cautious conclusion of this assessment is that environmental noise minimally helped the neural network to classify the valence and context of pig vocalizations.

As a final note, it can be seen from the t-SNE plots that a few experiments by the recording teams which were designed to record vocalizations from different contexts actually share single clusters. For example, Handling, Restrain, and Waiting recorded by INRA in a slaughterhouse environment are inarguably a single cluster in Figure 2b, and across all other tested perplexities (10-50). The same is the case for NegativeConditioning and PositiveConditioning, recorded by FBN. At first glance this might appear to represent that the neural network recognized these contexts by recording team rather than unique vocalization type. However, the neural network trained only on the set of vocalizations provided by INRA or FBN also had great difficulty distinguishing between their contexts. It is therefore probable that some of the experiments encapsulated in the collaborative dataset may not have been distinct enough to capture truly disparate vocalizations from the animals. This information may be useful to future researchers in devising their experimental protocols.

**Supplementary Figures**

**Supplementary Figure S1.** **Effect of the context category on the duration (Dur) for low-frequency calls (LF, above) and high-frequency calls (HF, below).** Model estimates, along with the lower and upper 95% confidence intervals of Dur (s) extracted from linear mixed-effect models. Context categories (see Supplementary Table S1 for the description of the contexts) are ordered according to the model estimate values (from the lowest to the highest duration) and the corresponding assumed valence is indicated in brackets (white (+) = positive; grey (-) = negative).

**Supplementary Figure S2.** **Effect of the context category on the amplitude modulation rate (AmpModRate) for low-frequency calls (LF, above) and high-frequency calls (HF, below).** Model estimates, along with the lower and upper 95% confidence intervals of AmpModRate (s^-1^) extracted from linear mixed-effect models. Context categories (see Supplementary Table S1 for the description of the contexts) are ordered according to the model estimate values (from the lowest to the highest AmpMod) and the corresponding assumed valence is indicated in brackets (white (+) = positive; grey (-) = negative).

**Supplementary Figure S3.** **Effect of the context category on the spectral centre of gravity (Q50%) for low-frequency calls (LF, above) and high-frequency calls (HF, below).** Model estimates, along with the lower and upper 95% confidence intervals of Q50% (Hz) extracted from linear mixed-effect models. Context categories (see Supplementary Table S1 for the description of the contexts) are ordered according to the model estimate values (from the lowest to the highest frequency) and the corresponding assumed valence is indicated in brackets (white (+) = positive; grey (-) = negative).

**Supplementary Figure S4.** **Effect of the context category on the Wiener entropy (WienEntropy) for low-frequency calls (LF, above) and high-frequency calls (HF, below).** Model estimates, along with the lower and upper 95% confidence intervals of WienEntropy extracted from linear mixed-effect models. Context categories (see Supplementary Table S1 for the description of the contexts) are ordered according to the model estimate values (from the lowest to the highest value, indicating more noisy calls) and the corresponding assumed valence is indicated in brackets (white (+) = positive; grey (-) = negative).


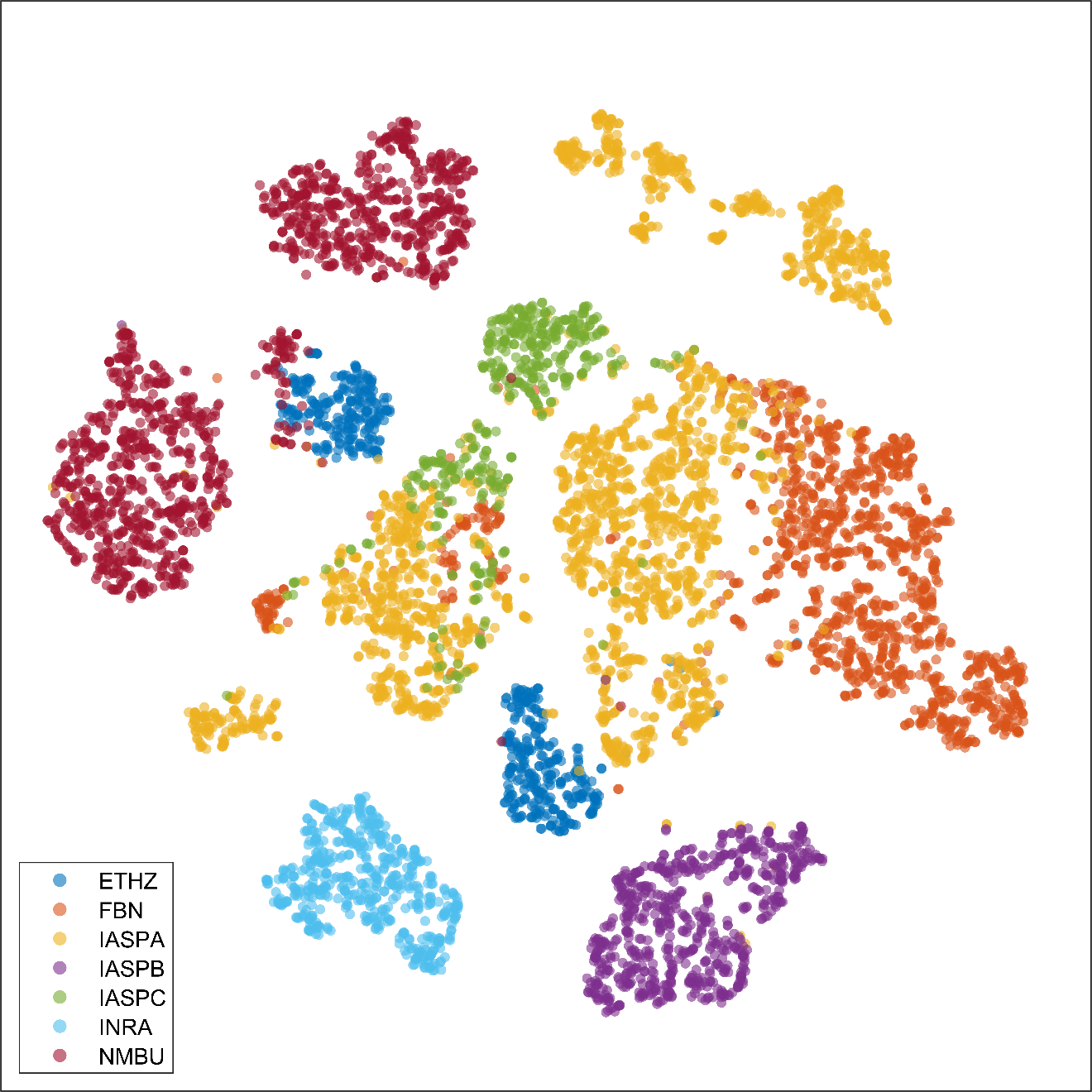


**Supplementary Figure S5.** t-SNE embedding of context classifying neural network’s last fully connected layer activations for each spectrogram, colored by recording team. This is the same t-SNE embedding as Figure 2b, but the points are colored by the team that recorded the associated vocalization.

**Supplementary Tables**

**Supplementary Table S1.** Recording contexts and attributed valence. List of the recording contexts and categories we studied according to their attributed valence, along with the following information: number of calls analyzed, number of animals recorded, description of the context, recording unit (i.e. whether the calls could be attributed to each individual (‘Ind’), pair or group (‘Grp’) of pigs), age class (1 = 1-25 days old; 2 = 32-43 days old; 3 ≥ 85 days old), breed (LW x L = Large White x Landrace; GL = German Landrace; TDLZL = Noroc), sex (M = Male; F = Female), recording team (IASP = Institute of Animal Science, Czechia; ETH = ETH Zurich, Switzerland; NMBU = Norwegian University of Life Sciences, Norway; FBN = Leibniz Institute for Farm Animal Biology, Germany; ETRE/INRAE = Bureau ETRE and French Research Institute for Agriculture, Food and Environment, France), reference providing further details about the recordings (published paper or Supplementary Text), and original name of the context in the provided reference.

| **Assumed valence** | **Context category** | **Nb calls** | **Nb pigs/ pairs/ groups** | **Context and description** | **Recording unit** | **Age class** | **Breed** | **Sex** | **Team** | **Reference** | **Original name** |
| --- | --- | --- | --- | --- | --- | --- | --- | --- | --- | --- | --- |
| **Positive** | Huddling | 74 | 11 | Huddling together with littermates | Ind | 1 | LW X L | M/F | IASP | ^5^ | Huddling |
|  | After Nursing | 79 | 10 | Approaching the head of the mother after nursing | Ind | 1 | LW X L | M/F | IASP | ^5^ | After nursing |
|  | Enriched | 337 | 39 | Exposition by pair in a familiar enriched arena for 5 min | Ind | 2 | LW | M/F | ETH | ^12^ | Positive |
|  |  |  |  | Exposition by pair in a familiar enriched arena for 10 min | Pair | 3 | TDLZL | M/F | NMBU | Sup Text | NA |
|  | Positive Conditioning | 119 | 20 | Exposition alone to a positively conditioned (familiar) arena for 10 min | Ind | 2 | GL | F | FBN | ^13^ | Positive conditioning |
|  | Before Nursing | 26 | 2 | Approaching the head of the mother before nursing | Ind | 1 | LW X L | M/F | IASP | ^5^ | Before nursing |
|  | Reunion | 945 | 85 | Reunion with mother and litter mates after 3 min of isolation | Ind | 1 | LW X L | M/F | IASP | Sup Text | NA |
|  |  |  |  | Reunion with mother and litter mates after 8 min of social isolation | Ind | 1 | LW X L | M/F | IASP | Sup Text | NA |
|  |  |  |  | Reunion with mother and litter mates after 10 min of social isolation | Ind | 1 | LW X L | M/F | IASP | ^5^ | Reunion |
|  | Running | 705 | 32 | Running by pair from house pen to a familiar arena | Pair | 3 | TDLZL | M/F | NMBU | Sup Text | NA |
|  |  |  |  | Running by pair from a familiar arena to house pen | Pair | 3 | TDLZL | M/F | NMBU | Sup Text | NA |
| **Negative** | Missed Nursing | 45 | 3 | Struggling to reach teats for nursing | Ind | 1 | LW X L | M/F | IASP | ^5^ | Missed nursing |
|  | Surprise | 17 | 10 | Being surprised by the arrival of a person | Ind | 1 | LW X L | M/F | IASP | ^5^ | Surprise |
|  | Barren | 588 | 16 | Exposition by pair in a familiar barren arena for 10 min | Pair | 3 | TDLZL | M/F | NMBU | Sup Text | NA |
|  | Novel Object | 333 | 20 | Exposition alone to a novel object in a novel arena for 5 min | Ind | 2 | GL | F | FBN | ^14^ | Novel object |
|  | Negative Conditioning | 119 | 20 | Exposition alone to a negatively conditioned (familiar) arena for 10 min | Ind | 2 | GL | F | FBN | ^13^ | Negative conditioning |
|  | Isolation | 2069 | 159 | Social isolation in a familiar arena for 3 min | Ind | 2 | LW | M/F | ETH | ^12^ | Isolation |
|  |  |  |  | Social isolation in a small box for 3 min | Ind | 1 | LW X L | M/F | IASP | Sup Text | NA |
|  |  |  |  | Social isolation in a novel arena for 5 min | Ind | 2 | GL | F | FBN | ^14^ | Open field |
|  |  |  |  | Social isolation in a small box for 8 min | Ind | 1 | LW X L | M/F | IASP | Sup Text | NA |
|  |  |  |  | Social isolation in a small pen for 10 min | Ind | 1 | LW X L | M/F | IASP | ^5^ | Isolation |
|  |  |  |  | Social isolation in an arena for 10 min | Ind | 2 | GL | F | FBN | ^14^ | Social isolation |
|  | Fighting | 57 | 4 | Fighting for teats during nursing | Ind | 1 | LW X L | M/F | IASP | ^5^ | Fighting |
|  | Crushing | 146 | 10 | Pressed against the floor while laterally lying with both hands to simulate crushing | Ind | 1 | LW X L | M/F | IASP | ^5^ | Crushing |
|  | Physical restrain/ holding | 1081 | 128 | Restrain in the arms of a stockperson for 1 min | Ind | 1 | LW X L | M/F | IASP | ^5^ | Arms of a person |
|  |  |  |  | Backtest, i.e. being turned and kept on the back with foreleg and belly held for X min | Ind | 1 | LW X L | M/F | IASP | ^1^ | Backtest |
|  |  |  |  | Sham castration, i.e. being restrained but the incision is not done, only simulated with blunt side of scalpel | Ind | 1 | LW X L | M | IASP | Sup Text | NA |
|  |  |  |  | Sham fixation as during castration | Ind | 1 | LW X L | M | IASP | Sup Text | NA |
|  |  |  |  | Introduction into the restraining device of the slaughterhouse | Grp | 3 | Unknown | M/F | ETRE/ INRAE | Sup Text | NA |
|  |  |  |  | Restraint with help of a dog harness in a restraint stand for 5 min | Ind | 2 | GL | M | FBN | ^15^ | Restraint |
|  | Castration | 295 | 23 | Castration without *anaesthetics* (being held and cut by a stockperson) | Ind | 1 | LW X L | M | IASP | ^5^ | Castration |
|  |  |  |  | Cutting | Ind | 1 | LW X L | M | IASP | Sup Text | NA |
|  |  |  |  | Fixing | Ind | 1 | LW X L | M | IASP | Sup Text | NA |
|  |  |  |  | Shearing | Ind | 1 | LW X L | M | IASP | Sup Text | NA |
|  |  |  |  | Treating | Ind | 1 | LW X L | M | IASP | Sup Text | NA |
|  | Handling in slaughterhouse | 301 | 19 | Painful handling in slaughterhouse, i.e. handling in an individual slaughter corridor using an electrical prod by an unfamiliar human | Grp | 3 | unknown | M/F | ETRE/ INRAE | Sup Text | NA |
|  |  |  |  | Not painful handling in slaughterhouse, i.e. handling in an individual slaughter corridor using voice and/or hand by an unfamiliar human | Grp | 3 | unknown | M/F | ETRE/ INRAE | Sup Text | NA |
|  | Waiting in slaughterhouse | 78 | 13 | Waiting in an individual slaughter corridor without human presence | Grp | 3 | unknown | M/F | ETRE/ INRAE | Sup Text | NA |

**Supplementary Table S2.** Number of calls of each type (LF = low-frequency calls; HF = high-frequency calls) used for the analyses, according to the attributed valence and context category (see Supplementary Table S1 for the description of the contexts).

| **Attributed valence** | **Context category** | **Call Type** | |  |
| --- | --- | --- | --- | --- |
|  |  | LF | HF | **TOTAL** |
| **Positive** | Huddling | 73 | 1 | **74** |
|  | After Nursing | 74 | 5 | **79** |
|  | Enriched | 333 | 4 | **337** |
|  | Positive Conditioning | 119 | 0 | **119** |
|  | Before Nursing | 23 | 3 | **26** |
|  | Reunion | 750 | 195 | 945 |
|  | Run from and to an arena | 688 | 17 | 705 |
|  | **Total** | **2060** | **225** | **2285** |
| **Negative** | Missed Nursing | 42 | 3 | **45** |
|  | Surprise | 17 | 0 | **17** |
|  | Barren | 584 | 4 | **588** |
|  | Novel Object | 206 | 127 | **333** |
|  | Negative Conditioning | 119 | 0 | **119** |
|  | Isolation | 1699 | 370 | **2069** |
|  | Fighting | 21 | 36 | **57** |
|  | Crushing | 78 | 68 | **146** |
|  | Physical restrain/holding | 468 | 613 | **1081** |
|  | Castration without anesthetics | 58 | 237 | **295** |
|  | Handling in slaughterhouse | 120 | 181 | **301** |
|  | Waiting in slaughterhouse | 41 | 37 | **78** |
|  | **Total** | **3453** | **1676** | **5129** |

**Supplementary Table S3. Selection of the acoustic parameters.** Loadings of the acoustic parameters (see Table 1 for the parameter abbreviation and description) on the principal components (PCs) with an eigenvalue greater than 1, extracted from the Principal Component Analyses carried out on each call type separately (LF = low-frequency calls; HF = high-frequency calls). For each PC and each category of parameters, the highest loading among loadings with r >= l0.5l is indicated in bold. For the categories containing several parameters, the parameter selected for further analyses (indicated in bold) was the one obtaining the highest loading the most often across PCs and call types.

|  |  | **LF** | | | |  | **HF** | | |
| --- | --- | --- | --- | --- | --- | --- | --- | --- | --- |
|  | **Parameter** | **PC1** | **PC2** | **PC3** | **PC4** |  | **PC1** | **PC2** | **PC3** |
| Duration | **Dur** | 0.407 | -0.273 | **-0.681** | 0.092 |  | 0.163 | **-0.726** | 0.010 |
| AM | AmpVar | 0.059 | 0.407 | **-0.755** | -0.309 |  | **0.533** | 0.475 | -0.013 |
|  | **AmpModRate** | **0.629** | -0.451 | -0.433 | 0.202 |  | -0.284 | **-0.773** | -0.266 |
|  | AmpModExtent | -0.504 | **0.680** | -0.159 | -0.296 |  | 0.350 | 0.768 | 0.249 |
| Spectrum | Q25% | -0.855 | 0.099 | -0.200 | 0.282 |  | 0.835 | -0.275 | 0.223 |
|  | **Q50%** | **-0.910** | -0.158 | -0.117 | 0.235 |  | **0.880** | -0.256 | 0.253 |
|  | Q75% | -0.759 | **-0.512** | 0.016 | -0.092 |  | 0.784 | -0.223 | 0.116 |
|  | FPeak | -0.706 | 0.144 | -0.249 | 0.424 |  | 0.731 | -0.196 | 0.254 |
| Noise | Harmonicity | -0.288 | -0.182 | -0.022 | **-0.685** |  | 0.577 | 0.153 | -0.709 |
|  | **WienEntropy** | **0.525** | **0.602** | 0.127 | 0.365 |  | **-0.610** | -0.102 | **0.711** |

**Supplementary Table S4.** Accuracy of CNN classifier (trained on vocalizations from every recording team) when classifying data from only one group at a time. To calculate these statistics, a neural network was trained on the given classification task for 20 epochs. The best accuracy model (as assessed by the validation set) produced during this training session was saved. This process was repeated 10 times for each classification task (valence/context). From the 10 trials, the mean accuracy is listed. The overall accuracy is the weighted average of the accuracies within each site.

| **Recording site** | **Valence** | **Context** |
| --- | --- | --- |
| ETHZ | 0.860 ± 0.007 | 0.893 ± 0.008 |
| FBN | 0.853 ± 0.008 | 0.623 ± 0.005 |
| IASPA | 0.943 ± 0.005 | 0.878 ± 0.004 |
| IASPB | 0.979 ± 0.004 | 0.983 ± 0.003 |
| IASPC | 0.989 ± 0.004 | 0.662 ± 0.015 |
| INRA | 0.902 ± 0.010 | 0.584 ± 0.015 |
| NMBU | 0.888 ± 0.007 | 0.913 ± 0.005 |
| **Total weighted accuracy** | **0.913 ± 0.006** | **0.815 ± 0.006** |

**Supplementary Table S5.** Accuracy of CNN classifiers trained on data from only one recording site. To calculate these accuracies, a neural network was trained on the given classification task for 20 epochs (e.g. classify the valence or context of calls from ETHZ). The best accuracy model (as assessed by the validation set) produced during this training session was saved. This process was repeated 10 times for each classification task (valence/context). From the 10 trials, the mean accuracy is listed. Some boxes show an accuracy of 1.000 ± 0.000 because there was only a single class label for the given data (i.e. within IASPB’s recordings, the vocalizations were all of positive valence and reunion context).

| **Recording site** | **Valence** | **Context** |
| --- | --- | --- |
| ETHZ | 0.901 ± 0.006 | 0.907 ± 0.007 |
| FBN | 0.919 ± 0.002 | 0.643 ± 0.005 |
| IASPA | 0.974 ± 0.001 | 0.894 ± 0.003 |
| IASPB | 1.000 ± 0.000 | 1.000 ± 0.000 |
| IASPC | 1.000 ± 0.000 | 0.708 ± 0.012 |
| INRA | 1.000 ± 0.000 | 0.608 ± 0.006 |
| NMBU | 0.940 ± 0.002 | 0.925 ± 0.002 |
| **Total weighted accuracy** | **0.958 ± 0.002** | **0.833 ± 0.004** |

Dataset S1 (separate file). Vocal parameters extracted from the calls and used in the linear mixed-effects models and permuted discriminant function.

**Supplementary References**

1. Linhart, P., Ratcliffe, V. F., Reby, D. & Špinka, M. Expression of emotional arousal in two different piglet call types. *PLoS ONE* **10**, e0135414 (2015).

2. Correa, J. A. *et al.* Effects of different moving devices at loading on stress response and meat quality in pigs. *J. Anim. Sci.* **88**, 4086–4093 (2010).

3. Charlton, B. D., Zhihe, Z. & Snyder, R. J. Vocal cues to identity and relatedness in giant pandas (*Ailuropoda melanoleuca*). *J. Acoust. Soc. Am.* **126**, 2721–2732 (2009).

4. Beckers, G. J. L. *Wiener entropy*. (2004).

5. Tallet, C. *et al.* Encoding of situations in the vocal repertoire of piglets (Sus scrofa): A comparison of discrete and graded classifications. *PLoS ONE* **8**, e71841 (2013).

6. Hartig, F. *DHARMa: Residual Diagnostics for Hierarchical (Multi-Level / Mixed) Regression Models* *[R package]*. *https://cran.r-project.org/web/packages/DHARMa* (2018).

7. Halekoh, U. & Højsgaard, S. A Kenward-Roger approximation and parametric bootstrap methods for tests in linear mixed models: The R package pbkrtest. *J. Stat. Softw.* **1**, 9 (2014).

8. Bates, D., Maechler, M., Bolker, B., Walker, S., Christensen, R. H. B., Singmann, H., Dai, B., Scheipl, F. & Grothendieck, G. *lme4: Linear mixed-effects models using S4 classes [R package]*. *http://CRAN.R-project.org/package=lme4* (2011).

9. Nakagawa, S. & Schielzeth, H. A general and simple method for obtaining R2 from generalized linear mixed-effects models. *Methods Ecol. Evol.* **4**, 133–142 (2013).

10. Mundry, R. & Sommer, C. Discriminant function analysis with nonindependent data: consequences and an alternative. *Anim. Behav.* **74**, 965–976 (2007).

11. Jespersen, C. K. *et al.* An Unambiguous Separation of Gamma-Ray Bursts into Two Classes from Prompt Emission Alone. *Astrophys. J. Lett.* **896**, L20 (2020).

12. Briefer, E. F., Vizier, E., Gygax, L. & Hillmann, E. Expression of emotional valence in pig closed-mouth grunts: Involvement of both source- and filter-related parameters. *J. Acoust. Soc. Am.* **145**, 2895 (2019).

13. Leliveld, L. M. C., Düpjan, S., Tuchscherer, A. & Puppe, B. Behavioural and physiological measures indicate subtle variations in the emotional valence of young pigs. *Physiol. Behav.* **157**, 116–124 (2016).

14. Leliveld, L. M. C., Düpjan, S., Tuchscherer, A. & Puppe, B. Vocal correlates of emotional reactivity within and across contexts in domestic pigs (*Sus scrofa*). *Physiol. Behav.* **181**, 117–126 (2017).

15. Leliveld, L., Düpjan, S., Tuchscherer, A. & Puppe, B. Hemispheric specialization for processing the communicative and emotional content of vocal communication in a social mammal, the domestic Pig. *Front. Behav. Neurosci.* **14**, 217 (2020).
